# The unfinished health agenda: Neonatal mortality in Cambodia

**DOI:** 10.1371/journal.pone.0173763

**Published:** 2017-03-21

**Authors:** Rathmony Hong, Pauline Yongeun Ahn, Frank Wieringa, Tung Rathavy, Ludovic Gauthier, Rathavuth Hong, Arnaud Laillou, Judit Van Geystelen, Jacques Berger, Etienne Poirot

**Affiliations:** 1 Child Survival and Development Section, UNICEF, Phnom Penh, Cambodia; 2 Multi-lateral Cooperation, KOICA Cambodia, Phnom Penh, Cambodia; 3 JRU Nutripass IRD-SupAgro-UM, Institut de Recherche pour le Developement, Montpellier, France; 4 Maternal Newborn Child Health Center, Ministry of Health, Phnom Penh, Cambodia; 5 International Health and Development, ICF, Rockville, Maryland, United Stataes of America; 6 Independent Consultant, Phnom Penh, Cambodia; Centre Hospitalier Universitaire Vaudois, FRANCE

## Abstract

**Background:**

Reduction of neonatal and under-five mortality rates remains a primary target in the achievement of universal health goals, as evident in renewed investments of Sustainable Development Goals. Various studies attribute declines in mortality to the combined effects of improvements in health care practices and changes in socio-economic factors. Since the early nineties, Cambodia has managed to evolve from a country devastated by war to a nation soon to enter the group of middle income countries. Cambodia's development efforts are reflected in some remarkable health outcomes such as a significant decline in child mortality rates and the early achievement of related Millennium Development Goals. An achievement acknowledged through the inclusion of Cambodia as one of the ten fast-track countries in the Partnership for Maternal, Newborn and Child Health. This study aims to highlight findings from the field so to provide evidence for future programming and policy efforts. It will be argued that to foster further advances in health, Cambodia will need to keep neonatal survival and health high on the agenda and tackle exacerbating inequities that arise from a pluralistic health system with considerable regional differences and socio-economic disparities.

**Methods/Findings:**

Data was drawn from Demographic Health Surveys (2000, 2005, 2010, 2014). Information on a series of demographic and socio-economic household characteristics and on child anthropometry, feeding practices and child health were collected from nationally representative samples. To reach the required sample size, live-births that occurred over the past 10 years before the date of the interview were included. Demographic variables included: gender of the child, living area (urban or rural; four ecological regions (constructed by merging provinces and the capital), mother’s age at birth (<20, 20–35, 35+), birth interval (long, short) and birth order (1st, 2–3, 4–6, 7+). Socio-economic variables included: mother education level (none, primary, secondary+) and household wealth (asset-based index). Data on antenatal care, tetanus injection and skilled assistance at birth were used for the mother's last child.

Between 2000 and 2014, Cambodia achieved a considerable reduction in neonatal mortality (46% reduction rate). By 2014, gender inequities became almost non-existent (for all measures of equality); inequity related to mother’s education decreased for all time periods; improvements were observed for differences in neonatal mortality by preceding birth interval; and a reduction in neonatal mortality rates could be noted among all the regional subgroups. Inequities increased between mothers who had limited antenatal care and those who received more than four antenatal care visits. In most scale indicators, the Slope Index of Inequality and Relative Index of Inequality estimates for all four rounds of the survey suggest inequity exacerbated in deprived communities. Also, wealth and residence (urban/rural divide) continued to be major determinants in neonatal mortality rates and related inequity trends.

**Conclusion:**

Analysis highlighted some of the complex patterns and determinants of neonatal mortality, in Cambodia. There has been a considerable decline in neonatal mortality which echoes global trends. Our analysis reveals that despite these advances, additional socio-economic and demographic characteristics considerably affected neonatal mortality rates and its inequities. There continue to be pockets of vulnerable groups that are lagging behind. This analysis highlights the determinants along the urban-rural and rich-poor divides in neonatal mortality inequities and how these affect access to and utilization of quality basic health services. This calls for future policy and programming efforts to be deliberate in their equity approach. Quality improvements in health services and targeted interventions for specific socio-economic groups will be required to further accelerate progress in reducing neonatal mortality and address Cambodia’s pressing unfinished agenda in health.

## Introduction

Infant and under-five mortality are indicators of overall child health in a population, whereas neonatal mortality is reflective of the quality of perinatal care to ensure effective delivery of essential maternal and newborn services [1]. Reducing mortality and improving the health status of young children are important milestones for governments and the international community. Under-five mortality was one of the eight Millennium Development Goals (MDGs) adopted by the United Nations (UN) Member States, in 2000. With the advent of the Sustainable Development Goals (SDG), newborn and under-five mortality continue to be primary targets to achieve universal health goals [2].

Although under-five mortality rates (U5MR) and neonatal mortality rates (NMR) have declined globally, from 91 and 36 to 43 and 19 per 1,000 live births between 2000 and 2015 respectively, the degree of progress varies across countries and regions. In South-East Asia, U5MR declined from 72 to 27 per 1,000 live births during the period 1990–2015, where NMR declined from 28 to 13 per 1,000 live births during the same time span [3] [4]. Changes in the levels of infant and child mortality also differed by socio-economic status of the household and level of education of the mother and various studies attribute declines in mortality to the combined effects of improved health care practices and socio-economic advancements [5] [6] [7]. In Cambodia, data from Demographic and Health Surveys (DHS) show a substantial decline in mortality rates; yet, NMR remains high at 18 per 1,000 live births [4] [8] [9] [10]. Data on deaths is collected through the civil registration system and the health services system [11] [12]. While Cambodia's registration system develops data from population surveys are used to produce national estimates. DHS provide data on neo-natal deaths and a series of socio-economic determinants. Potential sampling and non-sampling errors are taken into account, including underreporting through pattern analysis.

This paper uses data from DHS (2000–2014) to analyse a series of maternal and child care interventions and socio-economic determinants in relation to NMR, in Cambodia. Improved insight in NMR and relevant socio-economic factors will lay the foundation to guide the development of targeted health programs to further reduce mortality and promote child and population health.

## Methods

### Data

We used data from Cambodia Demographic and Health Surveys (CDHS) conducted in 2000, 2005, 2010 and 2014 to collect information from a nationally representative sample on household demographic and socio-economic characteristics and on child anthropometry, child feeding practices and child health. Women aged 15–49 years responded to a specific questionnaire that comprised questions on the total number of children born to them and maternal birth history. Birth history questions included date of birth for every live birth, survival status, current age of surviving children and age at death for those deceased. Because neonatal mortality is a rare event, live births that occurred during the ten years prior to the date of interview were included in order reach an adequate sample size. CDHS were based on stratified samples selected at two stages, and each reporting domain was separated into rural and urban areas.

### Measurements

This paper focuses on neonatal mortality, which refers to children who were born alive, but died within the first 30 days of life. We estimated NMR and examined changes in its inequities over time between several subgroups of the population. Population subgroups were constructed according to different socio-economic, demographic and health intervention factors with potential effect on mortality of children. Demographic variables included gender of the child, living area categorized as urban or rural, and region which was constructed by merging provinces into Plain, Great Lake, Coastal and Plateau/Mountain and Phnom Penh (the capital). Mother’s age at birth was classified into three groups (<20 years old, 20–35, 35+) to capture teenage and older pregnancies. We represented preceding birth intervals as long and short intervals, the latter being the case when the previous child was born less than two years before the one in question. Also, the birth order of the child was categorized into four groups (first born, 2–3, 4–6, 7 or more). Among the socio-economic variables, the education level of the mother was categorized as “none” for women who never attended school, “primary” for women who attended some primary school without necessarily having completed it, and “secondary+” for women who attended some secondary school or more. Household wealth was estimated in the DHS with an asset-based index that combines information about ownership of consumer goods, housing quality and water and sanitation facilities. As such, each household’s wealth score represents its position in the wealth distribution relative to other households. This household wealth index was constructed using principal component analysis with assets that were consistently available over time [13]. We also investigated NMR inequities relative to the number of antenatal visits of the mother, disaggregated into three groups (none, 1–3, 4+). Since questions about antenatal care, tetanus injection and skilled assistance were asked for the last child only, the sample of last-born children was used to investigate these determinants.

### Statistical analysis

#### Estimation of neonatal mortality rates

We first estimated NMR within each indicator described above. To do so, we used a synthetic cohort life table approach to calculate the probabilities of dying for small age segments based on real mortality experience. These mortality probabilities were then combined to estimate more common and larger age segments. The small age segments considered are the same as those adopted in the DHS, namely: 0, 1–2, 3–5, 6–11, 12–23, 24–35, 36–47 and 48–50 months. In addition, confidence intervals of these estimations were assessed using a bootstrap method.

#### Assessment of inequalities in neonatal mortality

We examined socio-economic and demographic inequities in NMR using absolute and relative measures of inequality. Slope index of inequality (SII) was used to examine absolute socio-economic inequities in NMR. The relative index of inequality (RII) was used to examine relative socio-economic inequities for scale variables. For binary variables, absolute difference was used to calculate absolute inequality, while relative risk was used to calculate relative inequality.

We calculated the SII and RII by regressing neonatal mortality outcomes against an individual’s relative rank in the cumulative distribution of socio-economic position. The relative socio-economic ranks range from 1 (most deprived part of the population) to 0 (most advantaged part of the population). For the determinants mother age at birth and preceding birth interval, we chose to attribute the rank value of 1 to the youngest women and the shortest intervals and 0 to the oldest women and the longest intervals. Because neonatal mortality is a binary outcome we calculated SII using a logistic regression model and estimated average marginal effects on the risk scale [14]. Thus, the coefficient for the rank variable (i.e. the slope) represents the estimated difference in NMR between the bottom and the top of the socio-economic distribution. Similarly, we obtained the RII using a log-binomial regression model on the risk scale and the coefficient for the rank variable to show the proportionate relative risk in NMR across the distribution of socio-economic positions. These two models were also used to determine absolute and relative NMR inequalities across binary determinants.

#### Adjustment for potential confounding variables

We adjusted estimates to account for potential confounding effects. Confounding factors were investigated based on knowledge about the country and statistical reasoning. When a confounder was identified for one survey, we adjusted all the others for this confounder. Finally, quadratic terms were included in the model when we found a U-shaped relationship between NMR and a confounder.

#### Tracking changes in inequality

To summarise changes in absolute and relative inequities in NMR over time, we calculated the absolute mean annual changes of the inequality measure between the last survey and the three others. Trends were assessed calculating the mean annual change of the inequality measures for three time periods. For graphical purposes, we show annual changes in relative inequality on a log-scale because it allowed a symmetry around zero. All analyses took into account the sampling design (stratification, clustering, sampling weights) and were conducted using Stata (version 13.0) and R (version 3.3.1) software.

### Ethics

DHS were approved by the Cambodia National Ethical Committee for Health Research. The Institutional Review Board of ICF International, Inc. reviewed and approved the Demographic and Health Survey Projects during all phases and the CDHS were categorized under those approvals. The Institutional Review Board of ICF International complies with the United States Department of Health and Human Services regulations for the protection of human research subjects (45 CFR 46).

## Results

Overall, data were available for almost 70,000 children, including 2,308 neonatal deaths. It can be noted that the proportion of neonatal deaths varies greatly across the sub-samples of the four CDHS, ranging from 4.18 percent in the sample for 2000, to 2.05 percent in the sample for 2014 (Table 1). Since these proportions are small, wide confidence intervals were expected.

**Table 1:** Neonatal deaths, Cambodia (2000–2014).

Year survey	Number children	Number neonatal deaths	Percentage sample
2000	20,522	852	4.18%
2005	17,735	666	3.76%
2010	16,288	483	2.97%
2014	14,711	302	2.05%
**Total**	**69,256**	**2,308**	**3.33%**

In Cambodia, neonatal mortality considerably declined over the past 14 years (Table 2). In 2000, 1 in 26 babies born alive did not survive their first month. By 2014, this rate was 1 in 48 live births. This represents a 46 percent reduction rate. Table 2, shows a pace of reduction in NMR in line with global and regional neonatal mortality trends. Neonatal mortality rates (NMR) decreased slightly between the year 2000 and 2005, then more considerably with the strongest decline seen between 2010 and 2014. During the period 2000–2014, no significant difference in neonatal mortality could be observed by tetanus toxoid status before pregnancy or skilled attendant during delivery. Consequently, these determinants were not included in the inequality analysis.

**Table 2:** Rate estimates for neonatal mortality, Cambodia (2000–2014).

Background characteristic	Deaths per 1000 live births (95% C.I)							
	2000		2005		2010		2014	
**Gender**								
Female	34.1	(30.5–37.6)	30.0	(26.1–33.8)	26.7	(23.1–30.4)	20.1	(15.8–24.3)
Male	44.0	(39.3–48.7)	42.4	(38.1–46.8)	35.6	(30.5–40.7)	22.1	(17.4–26.7)
**Residence**								
Urban	27.0	(20.7–33.3)	28.6	(23.0–34.3)	11.1	(8.0–14.3)	9.6	(5.9–13.3)
Rural	40.9	(37.3–44.6)	37.4	(33.8–41.1)	34.9	(30.7–39.1)	22.9	(20–25.9)
**Region**								
Phnom Penh	14.0	(5.5–22.5)	24.4	(13.0–35.8)	8.3	(2.0–14.6)	13.1	(3.9–22.2)
Plain	43.0	(37.2–48.7)	39.6	(33.0–46.3)	33.6	(27.8–39.4)	23.0	(17.2–28.8)
Great Lake	40.9	(36.2–45.6)	33.3	(28.8–37.3)	30.7	(25.6–35.7)	19.0	(14.8–23.2)
Coastal	40.5	(30.5–50.5)	37.1	(28.0–46.2)	36.2	(24.4–48.0)	20.2	(12.8–27.6)
Plateau/Mountain	32.4	(27.1–37.7)	39.1	(33.0–45.3)	35.7	(28.7–42.6)	24.8	(19.5–30.1)
**Wealth quintile**								
Lowest	39.9	(34.0–45.9)	36.3	(30.1–42.5)	38.2	(30.7–45.7)	22.5	(16.0–29.0)
Second	41.2	(36.0–46.4)	41.4	(33.1–49.7)	37.7	(30.7–44.8)	26.9	(19.7–34.0)
Middle	43.0	(35.9–50.0)	45.0	(37.2–52.8)	30.8	(22.0–39.5)	23.2	(16.7–29.8)
Fourth	40.4	(33.0–47.8)	31.8	(24.7–38.9)	29.2	(18.4–40.0)	20.5	(13.1–27.9)
Highest	27.0	(19.4–34.5)	21.6	(14.4–28.9)	14.1	(8.9–19.3)	11.0	(5.6–16.3)
**Mother education**								
Lowest	42.2	(38.0–46.5)	41.5	(34.9–48.0)	35.0	(27.3–42.7)	22.4	(16.9–27.9)
Primary	39.9	(35.6–44.2)	37.4	(32.7–42.1)	34.0	(29.5–38.5)	22.0	(17.3–26.6)
Secondary +	26.5	(19.7–33.3)	23.1	(16.7–29.6)	20.3	(14.0–26.6)	18.5	(13.5–23.6)
**Mother age at birth**								
<20	45.2	(34.5–55.9)	48.2	(37.1–59.3)	49.9	(35.1–64.7)	20.0	(12.7–27.3)
20–35	37.5	(33.7–41.3)	33.1	(29.7–36.5)	24.3	(20.9–27.6)	19.1	(16.2–22.1)
>35	45.0	(34.4–55.6)	42.3	(33.5–51.1)	61.7	(48.5–74.8)	42.1	(29.7–54.5)
**Birth order**								
First born	39.4	(32.3–46.5)	37.2	(30.0–44.5)	31.1	(25.1–37.0)	18.6	(14.1–23.1)
2–3	37.2	(31.9–42.5)	31.0	(26.0–35.9)	25.7	(20.7–30.6)	17.8	(14.0–21.6)
4–6	34.7	(29.8–39.5)	34.2	(28.2–40.2)	34.2	(26.6–41.7)	22.1	(15.5–28.8)
7+	52.6	(43.6–61.6)	57.4	(45.4–69.4)	59.9	(42.5–77.4)	75.9	(43.4–108.4)
**Preceding birth interval**								
Short (<24)	62.6	(54.7–70.5)	71.4	(59.8–83.0)	57.8	(44.7–71.0)	42.4	(27.9–56.9)
Long (24+)	29.6	(26.4–32.9)	25.2	(22.1–28.3)	24.8	(20.7–28.9)	17.9	(12.7–19.6)
**Number of antenatal visit**								
0	27.7	(20.9–34.5)	21.5	(14.9–28.1)	46.5	(28.8–64.1)	51.0	(20.8–81.2)
1–3	21.0	(14.5–27.4)	18.6	(12.8–24.5)	22.1	(12.9–31.3)	16.2	(5.5–26.9)
4+	12.4	(2.1–22.7)	11.4	(5.5–17.3)	12.1	(7.5–16.7)	8.5	(5.2–11.9)
**Two or more tetanus injections before pregnancy**								
Yes	41.8	(35.6–47.9)	37.3	(31.4–43.2)	32.0	(27.2–36.7)	21.5	(16.6–26.4)
No	38.7	(34.9–42.4)	36.2	(30.7–41.8)	31.7	(26.4–37.0)	23.0	(16.6–29.5)
**Skilled assistance at birth**								
Yes	41.0	(34.3–47.6)	34.3	(28.3–40.3)	30.1	(25.2–35.0)	20.7	(16.9–24.4)
No	38.9	(34.3–43.4)	38.5	(32.8–44.3)	35.9	(29.5–42.3)	32.5	(20.9–44.2)
**Total**	**39.1**	**(36.1–42.2)**	**36.3**	**(32.6–39.9)**	**31.3**	**(27.2–35.4)**	**21.1**	**(17.9–24.3)**

Note: Number of antenatal visits, tetanus injection and skilled assistance at birth were computed only for the last birth.

**Fig 1:**
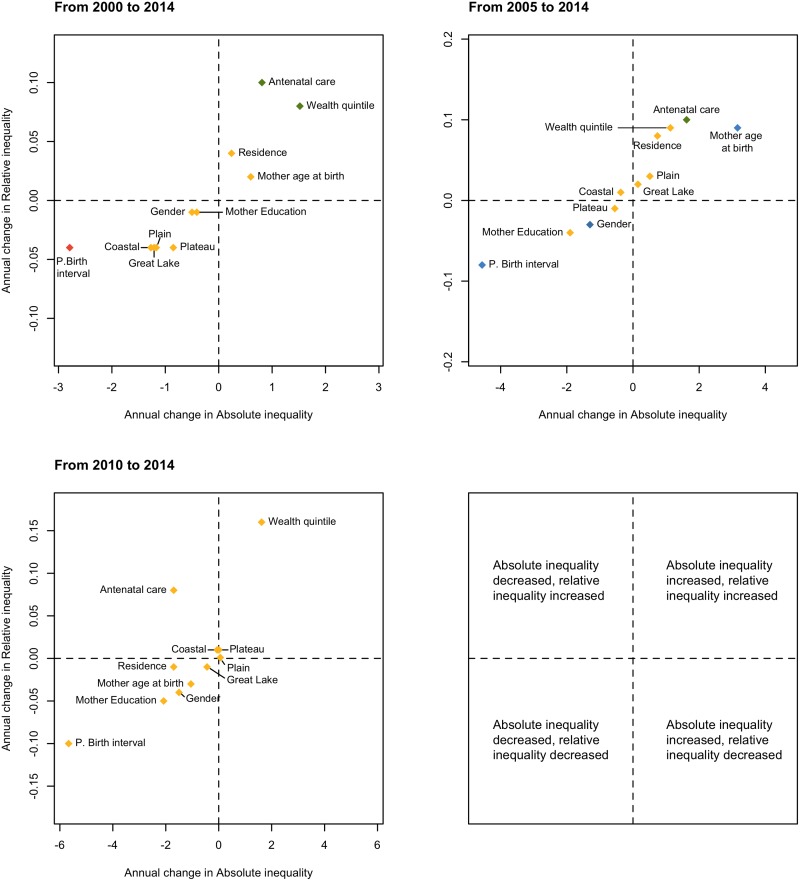
The association between mean annual changes in adjusted relative and absolute socio-economic inequities in meonatal mortality, Cambodia (2000–2014). (red) 95% CI excludes 0 for change in both SII and RII. (yellow) 95% CI includes 0 for change in both SII and RII. (green) 95% CI excludes 0 for change in RII only. (blue) 95% CI excludes 0 for change in SII only.

Table 3 provides the crude and adjusted estimations of inequality measures for each determinant while S1 Table (supporting information) gives an overview of trends in inequality. Crude and adjusted changes in absolute and relative inequality were plotted and are represented in Fig 1. This figure provides a snapshot of the evolution of inequities in neonatal mortality, in Cambodia, between 2000 and 2014. The adjusted estimations lead globally to the same direction of inequities than the crude ones, yet, the amplitude of the inequality gaps show systematically lower estimates (Table 3).

**Table 3:** Neonatal mortality inequality measures, Cambodia (2000–2014).

Binary outcome								
	Absolute Inequality: Absolute difference				Relative Inequality: Relative Risk			
	2000	2005	2010	2014	2000	2005	2010	2014
**Male vs Female**^1^								
Crude	10.0 (3.3, 16.7)	13.9 (6.8, 21.1)	8.4 (1.4, 15.5)	2.3 (-2.8, 7.4)	1.29 (1.09, 1.53)	1.46 (1.20, 1.78)	1.31 (1.04, 1.65)	1.11 (0.86, 1.41)
**Rural vs Urban**								
Crude	14.1 (6.1, 22.0)	9.5 (0.2, 18.7)	23.9 (18.1, 29.7)	13.2 (8.1, 18.4)	1.52 (1.16, 2.00)	1.33 (0.98, 1.81)	3.15 (2.17, 4.58)	2.39 (1.57, 3.62)
Adjusted^2^	9.9 (0.8, 18.9)	6.6 (-4.3, 17.5)	20.1 (12.2, 28.1)	13.3 (5.5, 21.1)	1.33 (0.99, 1.77)	1.21 (0.86, 1.70)	2.46 (1.52, 3.96)	2.42 (1.24, 4.71)
**Plain vs Phnom Penh**								
Crude	29.6 (19.0, 40.2)	16.8 (-3.3, 36.9)	25.8 (16.1, 35.4)	9.7 (-3.2, 22.7)	3.14 (1.65, 5.96)	1.69 (0.77, 3.71)	4.11 (1.78, 9.48)	1.75 (0.71, 4.33)
Adjusted^3^	28.1 (15.7, 40.4)	7.1 (-21.4, 35.6)	11.4 (-19.4, 42.3)	-11.7 (-44.1, 20.7)	2.89 (1.41, 5.91)	1.22 (0.51, 2.94)	1.58 (0.33, 7.62)	0.63 (0.22, 1.81)
**Great Lake vs Phnom Penh**								
Crude	26.6 (16.2, 37.0)	8.8 (-10.5, 28.1)	22.4 (13.4, 31.4)	6.3 (-5.8, 18.4)	2.92 (1.54, 5.55)	1.36 (0.63, 2.98)	3.70 (1.61, 8.52)	1.49 (0.61, 3.62)
Adjusted^3^	24.6 (13.2, 36.0)	6.4 (-16.7, 29.4)	9.5 (-11.6, 30.7)	-7.8 (-34.8, 19.3)	2.65 (1.36, 5.15)	1.24 (0.52, 2.99)	1.53 (0.48, 4.91)	0.68 (0.22, 2.11)
**Costal vs Phnom Penh**								
Crude	27.2 (13.1, 41.3)	14.1 (-8.3, 36.4)	27.7 (11.8, 43.7)	7.1 (-6.7, 20.9)	2.96 (1.49, 5.89)	1.58 (0.69, 3.64)	4.35 (1.75, 0.79)	1.55 (0.6, 4.01)
Adjusted^3^	26.3 (12.3, 40.3)	11.9 (-12.7, 36.5)	8.8 (-20.0, 37.6)	-8.6 (-30.3, 13.1)	2.86 (1.43, 5.75)	1.47 (0.59, 3.63)	1.55 (0.29, 8.32)	0.62 (0.22, 1.76)
**Plateau vs Phnom Penh**								
Crude	19.0 (7.3, 30.7)	15.0 (-4.9, 34.9)	27.0 (16.8, 37.1)	11.4 (-1.1, 23.8)	2.37 (1.21, 4.64)	1.62 (0.74, 3.55)	4.26 (1.84, 9.85)	1.88 (0.77, 4.55)
Adjusted^3^	18.5 (6.4, 30.6)	11.5 (-13.2, 36.3)	6.6 (-24.7, 37.9)	-6.6 (-34.6, 21.4)	2.32 (1.15, 4.69)	1.44 (0.57, 3.60)	1.31 (0.29, 5.81)	0.75 (0.25, 2.21)
**Scale variable**								
	**Absolute Inequality: SII**				**Relative Inequality: RII**			
**Wealth quintile**								
Crude	10.2 (-1.8, 22.1)	16.3 (3.2, 29.4)	25.9 (14.3, 37.4)	27.7 (9.7, 45.8)	1.30 (0.95, 1.77)	1.56 (1.08, 2.27)	2.31 (1.56, 3.41)	3.84 (1.65, 8.93)
Adjusted^2^	4.2 (-9.0, 17.4)	15.3 (1.2, 29.3)	19.0 (5.9, 32.0)	25.5 (6.0, 45.4)	1.11 (0.79, 1.56)	1,52 (1.02, 2.25)	1.84 (1.19, 2.84)	3.43 (1.36, 8.66)
**Mother education**								
Crude	12.4 (-1.0, 25.8)	20.6 (6.2, 34.9)	17.1 (3.6, 30.6)	6.3 (-5.0, 17.5)	1.37 (0.97, 1.92)	1.73 (1.19, 2.52)	1.70 (1.12, 2.57)	1.34 (0.79, 2.26)
Adjusted^4^	8.8 (-5.3, 22.9)	20.1 (5.2, 35.1)	11.3 (-3.1, 25.6)	3.0 (-7.7, 13.7)	1.25 (0.87, 1.79)	1.72 (1.15, 2.56)	1.43 (0.90, 2.26)	1.15 (0.69, 1.92)
**Mother age at birth**								
Crude	2.6 (-10.6, 15.7)	-3.5 (-17.7, 10.7)	12.9 (-3.6, 29.4)	14.4 (3.0, 25.8)	1.07 (0.76, 1.50)	0.90 (0.64, 1.27)	1.40 (0.82, 2.41)	1.78 (0.99, 3.21)
Adjusted^5^	-8.8 (-29.0, 11.4)	-28.8 (-51.7, -6.0)	4.6 (-18.6, 27.9)	0.4 (-14.6, 15.3)	0.79 (0.47, 1.34)	0.45 (0.25, 0.81)	1.16 (0.54, 2.52)	1.01 (0.48, 2.13)
**Preceding birth interval**^1^								
Crude	59.0 (43.4, 74.7)	60.9 (39.4, 82.5)	42.6 (23.0, 62.2)	19.9 (2.7, 37.1)	4.38 (3.07, 6.25)	4.70 (2.94, 7.52)	3.54 (2.11, 5.96)	2.38 (1.21, 4.69)
**Number of antenatal visit**								
Crude	16.9 (-2.7, 36.4)	14.8 (0.9, 28.7)	37.9 (16.4, 59.5)	26.6 (9.5, 43.7)	2.03 (0.91, 4.55)	2.31 (1.07, 5.00)	7.89 (2.87, 21.70)	9.90 (2.79, 35.10)
Adjusted^6^	9.7 (-12.6, 32.1)	6.4 (-9.4, 22.1)	27.8 (7.8, 47.8)	21.0 (5.2, 36.7)	1.51 (0.59, 3.85)	1.43 (0.59, 3.48)	4.57 (1.66, 12.59)	6.26 (1.82, 21.56)

Note: Number of antenatal visits was computed only for the last birth. Data in parentheses are 95% confidence interval. SII = Slope index of inequality. RII = Relative index of inequality.

^1^: No cofounders were found for this indicator, therefore, no adjusted estimations are provided;

^2^: Adjusted for Region;

^3^: Adjusted for Residence;

^4^: Adjusted for Residence, Mother age at birth, (Mother age at birth)2;

^5^: Adjusted for Residence, Birth order;

^6^: Adjusted for Birth order, Wealth.

Neonatal mortality declined considerably from 39.1 to 21.1 deaths per 1,000 living births between 2000 and 2014 (Table 2), with higher mortality rates among boys than among girls in each round of the survey. But the inequality in NMR between both genders narrowed considerably with the gap becoming almost non-existent by 2014. Indeed, while the absolute difference between male and female was of 10 and 13.9 neonatal deaths in 2000 and 2005 respectively, this disparity fell to 2.3 neonatal deaths by 2014 (Table 3). Finally, gender based inequality decreased in both, absolute and relative measures when considering the total time frame (2000–2014) (Fig 1).

A pattern of reduction in NMR was also found among residence subgroups. Between 2000 and 2014, NMR decreased by 64% for babies born to mothers living in urban areas and by 44% for those born to mothers living in rural areas (Table 2). Inequality measures show that rural births are more subject to neonatal mortality than urban ones. Nonetheless, while the absolute inequality gap between urban and rural area has remained nearly the same, the relative inequality seems to have increased. In 2014, the risk to die within the first month of life is 2.39 times more in babies born in rural households compared to those born in urban households (2.42 after adjustment for region). For 2000, this relative risk was estimated at 1.52 (1.33 when adjusted) (Table 3). It can be noted that the inequality gap increased considerably between 2005 and 2010, for both relative and absolute measures as well as crude and adjusted measures. During the period of 2010–2014, an overall decline in equality measures can be seen (most importantly for absolute crude inequality from 23.9 to 13.2 and least for relative adjusted inequality with values of 2.46 to 2.42). (Table 3, Fig 1).

In most scale indicators, the SII and RII estimates for every round of the survey suggest inequality disfavouring deprived communities in NMR (Table 3). For instance, the crude SII of wealth quintile in 2014 is 27.7 (25.5 after adjustment for region) indicating that moving from the top to the bottom of the wealth distribution is associated with 27.7 (25.5) more neonatal deaths per 1000 live births. Also, the corresponding RII is 3.84 (3.43 when adjusted for region), suggesting that babies born to households at the bottom of the wealth distribution are 3.84 (3.43) times more likely to die during their first month of life compared to those at the top. The inequality gap according to wealth of the households has progressively increased in both, absolute and relative measures for each time frame as shown in Fig 1, in both crude and adjusted estimations. The gap between the poorest and the richest constantly increased over the years; yet, the NMR within each population quintile has declined considerably, with 35% for the poorest group and 60% for the richest group, between 2000 and 2014 (Table 2).

The inequality gap in NMR according to mother’s education has decreased for each time period in both absolute and relative measures (Fig 1). For instance, the crude SII is divided by approximately two, between 2000 and 2014 and by almost three after adjustment for region (Table 3). A noticeable increase in the inequality gap was found between 2000 and 2005, where comparing the most educated mothers to the least is associated with roughly 20 more neonatal deaths per 1,000 live births for both crude and adjusted estimates. In 2014, the crude and adjusted SII for this determinant are respectively estimated at 6.3 and 3.0. Between 2000 and 2014, the NMR within the different mother education subgroups decreased, especially in the lowest and primary education groups (Table 2).

A noteworthy decrease is found in the gap observed for neonatal mortality by preceding birth interval. In 2000, babies born after the shortest interval had a 4.38 times higher risk to die within their first months compared to those born after the longest interval, RII fell to 2.38 by 2014. A similar decline is observed in the absolute inequality, whereby SII dropped progressively from 59 in 2000 to 19.9 in 2014.

The NMR according to mother’s age at child birth per subgroup followed an overall declining pattern from 2000 to 2014, except for the oldest subgroup (women > 35 years of age) for which NMR knew a limited decline from 45 to 42.1 for 2000 and 2014 respectively, with a remarkable increase between 2005 and 2010 from 42.3 to 61.7 respectively (Table 2).

Inequities between mothers who received minimal to no antenatal care and those who received four or more visits increased for all inequality measures (absolute and relative, crude and adjusted (birth order, wealth), between 2000 and 2014. Yet, for the last time frame (2010–2014) absolute inequality decreased (crude and adjusted) while a rise in relative inequality can be noted for both, crude and adjusted measures (Fig 1). In addition, an increase in NMR among mothers who never received antenatal care can be seen. However, this observation is subject to discussion as the associated confidence intervals are wide.

Finally, a reduction is noted in NMR among all the region’s subgroups, with an exception of Phnom Penh, the capital, for which the NMR is approximately the same for 2000 and 2014. This may be explained by the fact that the NMR was already low in the capital in 2000. For the four rounds of the survey, NMR in Phnom Penh is lower than the rates in the other regions (Table 2). Inequities between the capital and the other regions decreased, between 2000 and 2014 (Table 3, Fig 1), though small changes were recorded during the period 2005–2014 after adjustment for residence.

## Discussion

The results of this study show complex patterns in the determinants of neonatal mortality, in Cambodia, over time. Between 2000 and 2014, there has been an overall decline in neonatal mortality, which echoes global trends. Health sector interventions and socio-economic factors, both, seem to have an influence on NMR.

### Methodological considerations

The use of absolute and relative measures of inequality is important in providing a complete picture of inequities as these measures can lead to differing conclusions about the extent of change and differences in inequities [15]. The SII and RII are particularly adapted for variables that can be expressed on a scale to measure inequality across the entire distribution of socio-economic positions, in contrast to common measures that compare only extreme groups (e.g. the richest and poorest wealth quintiles).

### Urban-rural disparities

By 2030, according to forecasts from the Asian Development Bank, Cambodia’s urban population is expected to double, while the urban share of the national economy is estimated to rise to 70%. As the engine for growth becomes concentrated in its urban capital, Cambodia’s trends in access and utilization of health services have changed and inequities exacerbated. This pattern is clearly manifested in the analysis. Children in rural areas continue to be more at risk for neonatal mortality than those from urban areas. This confirms findings from a previous study using DHS data for 38 countries that the neonatal mortality rate was on average 20% greater among infants born in rural areas [16] and implies that access to basic quality maternal and child health services remains a concern in rural Cambodia. Considering that more than half of newborn deaths in low-income countries occur at home, true figures of neonatal deaths in rural areas may exceed that of published vital statistics. However, when singling out Phnom Penh to compare its relative risk for inequality compared with other regions there seems to be minimal difference in risk. This may point to the growing burden of slum populations within the city. Both, rural and urban poor must become a priority for future interventions.

### Wealth inequities

Household wealth turns out to be one of the strongest determinants of neonatal mortality, in Cambodia. Between 2000 and 2014, the inequity gap between the haves and the have-nots has progressively increased over time, even though absolute and relative measures of NMR declined for all income quintiles. This justifies an equity-based approach that targets the most disadvantaged populations for improved access and utilization of quality basic health interventions. A retrospective equity analysis conducted using data from 54 countdown countries between 2000 and 2008 supports our finding that poor households are at increased risk for neonatal mortality because they experience barriers to access and utilization [17]. The widest inequity gap was observed for services delivered in fixed-site services, such as, births attended by skilled medical personnel, followed by four or more antenatal care services. Interventions often delivered at the community level were more equitable [17]. Our results add to the existing literature that renewed efforts are required to provide quality services with an equity focus through the integration of mainstream equity goals into health policies and programs at national and subnational levels.

### Maternal care services (Prenatal care) in the continuum of care period

Antenatal care (ANC) visits, especially when exceeding four times, is frequently used as an indicator reflective of the quality of health services and client satisfaction. In Cambodia, ANC rates have markedly increased over the last decade whilst exacerbating regional inequity. The danger of disregarding prenatal services is paramount in the context of Cambodia. In 2014, NMR for mothers who did not receive any antenatal services was 6.26 times higher than for those who received 4 ANC visits or more, even after adjustment for potential confounders. A retrospective cohort study conducted in the US using vital statistics from 1995–2000 corroborates this finding, whereby inadequate prenatal care is associated with increased neonatal deaths, in both, the presence and absence of antenatal high-risk conditions (e.g. anemia, cardiac disease, lung disease, hypertension, previous preterm/small for gestational age at birth). Therefore, skipping prenatal care services can be life threatening for the child [18]. The association between inadequate prenatal care and neonatal mortality may be mediated by increased risk of preterm delivery and low birth weight babies in these pregnancies. This is consistent with figures from the three provinces. Provinces with the lowest ANC visits were also the ones that have the highest pre-term/low birth weight babies. The same provinces also exhibit the highest prevalence of teenage pregnancies (ages 15–19 years), a well-known factor of increased risk for adverse birth outcomes and neonatal mortality—independent of other confounders [19] The high prevalence of teenage pregnancies may be related to local traditional expectations of early marriage. The United Nations Population Fund (UNFPA) reports that rural Cambodian girls may face pressure to quit school to help their families with farming or domestic work and start their own family early.

Both, improved access and utilization of quality basic health care services, and adequate attention to relevant socio-economic factors, will be required to promote further accelerated progresses in the reduction of NMR in the country.

## Conclusion

This paper shows some of the complex patterns in determinants of neonatal mortality in Cambodia over a 15 year period of time. Between 2000 and 2014, there has been a considerable decline in neonatal mortality, which echoes global trends. Cambodia, selected as one of the five fast-track countries in the Partnership for Maternal, Newborn and Child Health has benefitted from substantial development support to extend and expand quality of maternal and child health interventions. It can be assumed that this support is positively reflected in the decrease of neonatal mortality and the early achievement of relevant MDGs [20]. Our analysis reveals that despite these advances in health sector development, an additional series of socio-economic and demographic characteristics considerably influence NMR and its inequities. Seemingly, there continue to be pockets of vulnerable groups that are lagging behind. This analysis highlights the importance of the urban-rural and poor-wealth divides in NMR inequities together with inequities in access to and utilization of quality basic health care interventions. This calls for future policy and programming efforts to be deliberate in their equity approach. Quality improvements in health services and targeted interventions for specific socio-economic groups will be required to further accelerate progress in reducing neonatal mortality and address Cambodia’s pressing unfinished agenda in health.

## Supporting information

S1 Table Absolute annual changes in absolute and relative inequalities for neonatal mortality for the 10 years period before the survey, by background characteristics (Cambodia, 2000–2014). Note: Absolute annual changes were calculated as the difference between the absolute value of the two rounds of the survey's estimations divided by the number of years between the two rounds of the survey in question. Number of antenatal visit was computed only for the last birth. Data in parentheses are 95% confidence interval. SII = Slope index of inequality. RII = Relative index of inequality. 1: No cofounders were found for this indicator, therefore no adjusted estimations are provided. 2: Adjusted for Region; 3: Adjusted for Residence; 4: Adjusted for Residence, Mother age at birth, (Mother age at birth)2; 5: Adjusted for Residence, Birth order; 6: Adjusted for Birth order and Wealth.
(DOCX)

## Abbreviations

ANC: Antenatal Care
CDHS: Cambodia Demographic and Health Survey
DHS: Demographic and Health Survey
MDGs: Millennium Development Goals
NMR: Neonatal Mortality Rates
RII: Relative Index of Inequality
SDGs: Sustainable Development Goals
SII: Slope Index of Inequality
U5MR: Under-five Mortality Rates
UN: United Nations
UNFPA: United Nations Population Fund
UNICEF: United Nations Children’s Fund
