# Supplementary material for: The unfinished health agenda: Neonatal mortality in Cambodia
Source: PLoS One. 2017 Mar 21;12(3):e0173763. doi: 10.1371/journal.pone.0173763 (PMC5360239; doi:10.1371/journal.pone.0173763)
Supplement: S1 Table — Note: Absolute annual changes were calculated as the difference between the absolute value of the two rounds of the survey's estimations divided by the number of years between the two rounds of the survey in question. Number of antenatal visit was computed only for the last birth. Data in parentheses are 95% confidence interval. SII = Slope index of inequality. RII = Relative index of inequality. 1: No cofounders were found for this indicator, therefore no adjusted estimations are provided. 2: Adjusted for Region; 3: Adjusted for Residence; 4: Adjusted for Residence, Mother age at birth, (Mother age at birth)2; 5: Adjusted for Residence, Birth order; 6: Adjusted for Birth order and Wealth. (DOCX) [file pone.0173763.s001.docx]

|  | | | | | | | |
| --- | --- | --- | --- | --- | --- | --- | --- |
| **Binary outcome** | | | | | | | |
|  | | **Absolute Inequality : Absolute difference** | | | **Relative Inequality : Relative Risk (Log scale)** | | |
|  |  | **2014-2000** | **2014-2005** | **2014-2010** | **2014-2000** | **2014-2005** | **2014-2010** |
| **Male vs Female^1^** | | | | |  | | |
| Crude | | -0.55 (-1.15 , 0.05) | -1.29 (-2.26 , -0.32) | -1.53 (-3.69 , 0.64) | -0.01 (-0.03 , 0.01) | -0.03 (-0.07 , 0.01) | -0.04 (-0.13 , 0.04) |
| **Rural vs Urban** | | | | |  | | |
| Crude | | -0.06 (-0.74 , 0.61) | 0.41 (-0.77 , 1.58) | -2.67 (-4.60 , -0.75) | 0.03 (-0.01 , 0.07) | 0.06 (0.01 , 0.12) | -0.07 (-0.21 , 0.07) |
| Adjusted**^2^** | | 0.24 (-0.61 , 1.10) | 0.74 (-0.74 , 2.23) | -1.70 (-4.49 , 1.09) | 0.04 (-0.01 , 0.09) | 0.08 (-0.01 , 0.16) | -0.01 (-0.21 , 0.20) |
| **Plain vs Phnom Penh** | | | | |  | | |
| Crude | | -1.42 (-2.61 , -0.23) | -0.79 (-3.44 , 1.86) | -4.03 (-8.06 , 0.01) | -0.04 (-0.12 , 0.04) | 0.01 (-0.13 , 0.14) | -0.21 (-0.52 , 0.09) |
| Adjusted**^3^** | | -1.17 (-3.65, 1.31) | 0.51 (-4.28 , 5.30) | 0.06 (-11.12 , 11.25) | -0.04 (-0.13 , 0.05) | 0.03 (-0.12 , 0.18) | 0.00 (-0.47 , 0.47) |
| **Great Lake vs Phnom Penh** | | | | |  | | |
| Crude | | -1.45 (-2.59 , -0.31) | -0.28 (-2.81 , 2.25) | -4.02 (-7.79 , -0.26) | -0.05 (-0.13 , 0.03) | 0.01 (-0.12 , 0.14) | -0.23 (-0.53 , 0.08) |
| Adjusted**^3^** | | -1.21 (-3.30 , 0.89) | 0.15 (-3.80 , 4.11) | -0.44 (-9.03 , 8.15) | -0.04 (-0.13, 0.05) | 0.02 (-0.14 , 0.18) | -0.01 (-0.42 , 0.40) |
| **Costal vs Phnom Penh** | | | | |  | | |
| Crude | | -1.44 (-2.84 , -0.03) | -0.78 (-3.69 , 2.14) | -5.15 (-10.41 , 0.01) | -0.05 (-0.13 , 0.04) | - 0.01 (-0.14 , 0.14) | -0.26 (-0.58 , 0.06) |
| Adjusted**^3^** | | -1.27 (-3.11 , 0.58) | -0.37 (-4.01 , 3.28) | -0.06 (-9.07 , 8.95) | -0.04 (-0.13 , 0.05) | 0.01 (-0.14 , 0.16) | 0.01 (-0.48 , 0.50) |
| **Plateau vs Phnom Penh** | | | | |  | | |
| Crude | | -0.54 (-1.76 , 0.68) | -0.40 (-3.01 , 2.21) | -3.90 (-7.93 , 0.13) | -0.02 (-0.10 , 0.06) | 0,02 (-0,11 , 0,15) | -0,20 (-0,51 , 0,10) |
| Adjusted**^3^** | -0.85 (-3.03 , 1.33) | | -0.55 (-4.70 , 3.60) | 0.01 (-10.49 , 10.49) | -0.04 (-0.13 , 0.05) | -0.01 (-0.16 , 0.15) | 0.01 (-0.45 , 0.46) |
| **Scale variable** | | | | | | | |
|  | **Absolute Inequality : SII** | | | | **Relative Inequality : RII** | | |
| **Wealth quintile** | | | | |  | | |
| Crude | 1.25 (-0.30 , 2.8) | | 1.27 (-1.21 , 3.74) | 0.45 (-4.91 , 5.81) | 0.08 (0.01 , 0.14) | 0.10 (0.00 , 0.20) | 0.13 (-0.11 , 0.36) |
| Adjusted**^2^** | 1.52 (-0.18 , 3.23) | | 1.13 (-1.58 , 3.84) | 1.62 (-4.33 , 7.58) | 0.08 (0.01 , 0.15) | 0.09 (-0.02 , 0.20) | 0.16 (-0.10 , 0.41) |
| **Mother education** | | | | |  | | |
| Crude | -0.44 (-1.69 , 0.82) | | -1.59 (-3.62 , 0.44) | -2.70 (-7.10 , 1.70) | 0.00 (-0.05 , 0.04) | -0.03 (-0.10 , 0.04) | -0.06 (-0.23 , 0.11) |
| Adjusted^4^ | -0.41 (-1.68 , 0.85) | | -1.90 (-3.95 . 0.15) | -2.08 (-6.55 , 2.40) | -0.01 (-0.05 , 0.04) | -0.04 (-0.12 , 0.03) | -0.05 (-0.22 , 0.12) |
| **Mother age at birth** | | | | |  | | |
| Crude | 0.84 (-0.40 , 2.09) | | 1.94 (-0.08 , 3.96) | 0.38 (-4.63 , 5.38) | 0.04 (-0.01 , 0.09) | 0.08 (0.00 , 0.15) | 0.06 (-0.14 , 0.26) |
| Adjusted^5^ | 0.60 (-1.19 , 2.40) | | 3.16 (0.13 , 6.20) | -1.05 (-7.96 , 5.86) | 0.02 (-0.05 , 0.08) | 0.09 (-0.02 , 0.19) | -0.03 (-0.30 , 0.23) |
| **Preceding birth interval^1^** | | | | |  | | |
| Crude | -2.79 (-4.45 , -1.13) | | -4.56 (-7.62 , -1.50) | -5.67 (-12.19 , 0.84) | -0.04 (-0.10 , 0.01) | -0.08 (-0.17 , 0.02) | -0.10 (-0.31 , 0.11) |
| **Number of antenatal visit** | | | | |  | | |
| Crude | 0.69 (-1.16 , 2.55) | | 1.31 (-1.14 , 3.76) | -2.78 ( -9.65 , 4.09) | 0.11 (0.01 , 0.22) | 0.16 (0.00 , 0.33) | 0.06 (-0.35 , 0.46) |
| Adjusted**^6^** | 0.81 (-1.15 , 2.76) | | 1.62 (-0.85, 4.10) | -1.70 (-8.07, 4.67) | 0.10 (-0.01 , 0.21) | 0.16 (0.00, 0.33) | 0.08 (-0.32 , 0.48) |
|  | | | | | | | |
